# Correlation between branched-chain amino acids intake and total lymphocyte count in head and neck cancer patients: a cross-sectional study

**DOI:** 10.1186/s40795-023-00746-5

**Published:** 2023-07-14

**Authors:** Yosua Yan Kristian, Rahmat Cahyanur, Yohannessa Wulandari, Wina Sinaga, Widjaja Lukito, Findy Prasetyawaty, Wiji Lestari

**Affiliations:** 1 Department of Nutrition, Faculty of Medicine, Universitas Indonesia – Dr Cipto Mangunkusumo General Hospital, Jakarta, Indonesia; 2 Department of Internal Medicine, Faculty of Medicine, Universitas Indonesia - Dr Cipto Mangunkusumo General Hospital, Jakarta, Indonesia

**Keywords:** Branched-chain amino acids, Total lymphocyte count, Head and neck cancer

## Abstract

**Background:**

Cellular immunity as reflected by total lymphocyte count (TLC) has been proven to be related to overall survival rate cancer patients. Lymphocyte proliferation is regulated, to some extent, by nutritional factor. Branched chain amino acid (BCAA) is documented as one of numerous nutrients that play important role in lymphocyte proliferation through its effect on protein synthesis and DNA replication. Many studies describe the correlation between BCAA and TLC in hepatic cancer patients. This study emphasized the observation of that links in head and neck cancer patients.

**Methods:**

Eighty-five subjects were included in final analysis, aged 18–75, mostly male, with head and neck cancer who had not received treatment participated in this cross-sectional study at the Dr. Cipto Mangunkusumo General Hospital’s radiation and medical haematology oncology clinic. The BCAAs intake was assessed using a semi-quantitative food frequency questionnaire. Flow cytometry method was used to quantify TLC.

**Results:**

Overall, the subjects’ nutritional status mostly was considered normal, with the median intake of 1505 (800–3040) kcal/day of energy and mean of 73.96 ± 23.39 g/day of protein. Moreover, subjects’ average BCAA intake was 10.92 ± 0.48 g/day. Meanwhile, 17.6% of subjects were found to have low TLC level. From thorough analysis, we did not find a strong correlation between BCAA level and TLC (r = 0.235, p = 0.056).

**Conclusion:**

In participants with head and neck cancer who had not received chemoradiotherapy, there is no correlation between BCAA intake and TLC. The contribution of non-BCAA amino acids from dietary sources to lymphocyte proliferation requires further investigation.

**Trial registration:**

Retrospectively registered, with clinical trial number NCT05226065 on February 7th 2022.

## Background

Cancer incidence was estimated at 18 million in 2018, and it is the second leading cause of death worldwide [1]. Cancer-related deaths increased from about 6 million in 2000 to more than 9 million in 2018[2]. The incidence of cancer will reach 28.4 million in 2040[3]. In Indonesia, cancer prevalence was 1.4 per 1000 population in 2013, and it had risen to 1.8 per 1000 population within five years [4]. Head and neck cancer is one of the most common cancers, with an incidence of almost 5% of total cancer incidence in 2020[3]. Head and neck cancers mostly arise from the upper aerodigestive tract, which consist of the oral cavity, nasopharynx, oropharynx, hypopharynx, larynx, and nose and paranasal sinuses [5].

Cancer prognosis is affected by several factors, such as environment, tumour-related, and host, which are important in patient management. Environmental factors are the treatments’ quality, access to healthcare providers, and drug availability which impact the outcome of cancer care. Tumour related factors are the cell pathology, anatomical location, and biological properties of the tumour [6]. Host factors include patients’ gender, age, ethnicity, immune status, existing comorbidities, performance and nutritional status [6, 7]. Low intake due to anorexia, xerostomia, malabsorption, diarrhoea, nausea, vomiting, teeth abnormality, and excruciating pain leads to lower immune status and malnutrition [8]. Patients with weight loss had a shorter survival and showed more anaemia cases during chemotherapy [9]. Furthermore, there is a misconception among Indonesian people that fish and red meat can worsen cancer patients’ conditions. Therefore, they tend to avoid red meat and fish.

The immune system plays a role in the cancer immunity cycle, where T cell responses are needed to eliminate cancer cells [10]. A higher lymphocyte signature was observed to give a better outcome to overall survival [11]. Huang et al. concluded that lower mortality was found in head and neck cancer patients with higher baseline lymphocyte counts, while there was no association between the survival with haemoglobin, lymphocyte-to-neutrophile ratio, age, gender, and race [12]. Lymphocyte, alongside albumin, is the component in the prognostic nutritional index (PNI), an indicator of immune and nutrition status [13]. Lower TLC increases the infection frequency and severity [14]. Either nutritional or immune status not only affects the prognosis of the patient but also influences the chemotherapy effectiveness and surgical risk [9, 14].

Lymphocyte regulation is affected by immune signals and metabolic cues, one of which is nutrition such as amino acids [15]. Adequate BCAA intake increases TLC and the immune status [16, 17]. Branched-chain amino acids consist of leucine, isoleucine, and valine, and have a function in protein synthesis and as nitrogen donors for the synthesis of other amino acids [18]. Branched-chain amino acids also can be oxidized by immune cells due to the ability to express branched-chain alpha-keto acid dehydrogenase and decarboxylase. The BCAA uptake in B cells was also studied, and the highest uptake is found during the S phase [19]. Further study indicates that leucine can affect the mammalian target of the rapamycin (mTOR) signalling pathway [15]. The mTOR pathway controls protein translation, cell growth and proliferation, which immune cells are sensitive to [20, 21]. Several in vitro studies show that lack of BCAA resulted in lymphocyte proliferation disturbance, while a higher concentration of plasma BCAA concentration gives a small effect on lymphocyte proliferation [22–24]. These results were also supported by animal and human studies [25, 26].

Some newer studies used BCAA supplementation as the main source for intake adequacy [16, 27]. Nojiri et al. compared BCAA supplementation to diet only in hepatocellular carcinoma patients, better outcomes in survival rate and fewer complications were found, with a higher TLC was also observed in supplemented subjects [27]. Branched-chain amino acids have shown to benefit hepatic cancer patients [28, 29]. Supplementations are generally more expensive than food sources; where financial barriers are commonly found during cancer therapy in which patients undergo a “financial toxicity”[30]. Further studies are still needed to conclude whether BCAA from food sources are correlated to TLC in cancer patients. We therefore conducted a cross-sectional study to evaluate the correlation between BCAA intake and TLC in head and neck cancer patients. We believed that there was a significant correlation between BCAA intake and TLC in the head and neck cancer patients.

## Methods

### Subjects and study design

A cross-sectional study was conducted to head and neck cancer patients between January to November 2021 in the radiotherapy outpatient unit Dr Cipto Mangunkusumo General Hospital, Jakarta, Indonesia. Subjects were recruited using consecutive sampling, with the inclusion criteria being (i) over 18 years old and (ii) not having chemoradiotherapy or having finished chemoradiotherapy. Subjects with immune suppressant drugs, infections, and immune-related diseases are excluded. A minimum sample size of 85 subjects was needed to calculate the correlation coefficient (α = 0.05, β = 0.1, r = 0.3). Subjects’ characteristics, which are age, gender, cancer histopathology, cancer location and stage, were taken from medical records within the same period. The cancer staging was based on American Joint Committee on Cancer (AJCC) classification, which covered tumour size, regional lymph node, and metastasis [5]. Subjects were classified into stage 0, stage I, stage II, stage III, and stage IV. A cut-off of 60 years old was used for older adults [31]. From the total of 87 patients who visited the outpatient unit and met the inclusion criteria, two patients were uncontactable further to assess the dietary intake follow-up, making a total of 85 participants.

This study was approved by the Research Ethics Committee, Faculty of Medicine, Universitas Indonesia and Dr Cipto Mangunkusumo General Hospital with protocol ID 20-08-0928 and ethical registration number KET-901/UN2.F1/ETIK/PPM.00.02/2020, and is registered at clinicaltrial.gov number NCT05226065. Oral and written consent was taken from the subjects before conducting the research.

### Anthropometry measurement

Body weight was measured using a SECA scale type 876 with weight measurement to the nearest 0.1 kg. A two-meter GEA Microtoise was used for measuring body height to the nearest 0.1 cm. Subjects were barefoot, stood erect and only wore light clothes during the body height measurement [32]. The anthropometric measurements were done twice, and the average results were used to calculate the body mass index (BMI). Asia Pacific BMI classification was used to determine the subject’s nutritional status [33]. Body mass index < 18.5 kg/m2 was defined as underweight, 18.5 to < 23 kg/m2 was normal, and BMI ≥ 23 kg/m^2^ was overweight and obese.

### Dietary assessment

All of the field enumerators were medical doctors who were taking a clinical nutrition specialization program at the Faculty of Medicine, Universitas Indonesia (FMUI), and experienced in assessing dietary intake. The training was given to the enumerators to ensure the use of standardized measurement and methods. The FMUI has been conducting a residency program in clinical nutrition for more than 10 years. Medical graduates who have fulfilled the requirements for residency training were instructed on core competence in dietary intake and body composition assessments; and they comply with the set rotating schedules to various clinical departments, aiming to fulfil their specialist skills. The dietary intake was conducted using a semi-quantitative food frequency questionnaire (SQ-FFQ) [34]. The food frequency questionnaire has a moderate relative validity compared to a 4-day weighed food record for protein and several food groups [35]. Subjects were asked about their usual eating habit during the last 30 days, and the results were quantified using a food exchange list by the Ministry of Health Republic Indonesia [36]. Branched-chain amino acids intake was analyzed by adding leucine, isoleucine, and valine using the United States Department of Agriculture (USDA) database from NutriSurvey 2007 software [32]. Data regarding the total energy intake, protein intake, and non-protein calorie intake was also taken during the study using the same SQ-FFQ, program, and database. Protein calorie was calculated with total protein intake multiplied by four, and non-protein calorie intake was taken with a calculation of total energy intake minus protein calorie [37]. Blinding was performed during collecting or processing data. We used three different field enumerators for each subject, one for interviewing the subjects, gathering data from medical records, and data cleaning. The identity of the subjects was not visible to the enumerator during processing the data.

### Blood sample collection

A one-time blood sample was taken at the hospital laboratory after the dietary assessment. A sample of 5 ml blood was collected from the peripheral vein, and the TLC was obtained using a Sysmex® haematology autoanalyzer. Low TLC was defined as less than 1,0 × 10^3^/µL [38].

### Statistical analysis

Kolmogorov-Smirnov and Shapiro-Wilk normality test was performed. Data was defined as normally distributed if p > 0.05. Independent T-test and Mann-Whitney was used to compare the means between two groups. Data between more than two categories were analyzed with ANOVA for normally distributed data with the same variance, and LSD post hoc analysis was performed afterwards. The significance level chosen was p < 0.05.

The correlations between BCAA intake, potential confounders, and TLC were performed using the Pearson test for data with normal distribution or using the Spearman test for data with non-normal distribution. The two-tailed significance was chosen with the level of significance at p < 0.05 for the dependent and independent variables. The correlation between potential confounders and TLC was performed with a significance level of p < 0.25. Potential confounders which had non-normal distribution, strong significant correlation, marked by a correlation coefficient > 0.8 with BCAA intake were excluded from linear regression analysis. Linear regression analysis was done between the BCAA intake, potential confounders, and TLC with a level of significance of p < 0.05.

## Results

The subjects’ characteristics are shown in Table 1. A total of 85 participants was analyzed in the study. Head and neck cancers were more prevalent in male subjects, and most of the subjects were under 60 years old, and with normal BMI. Squamous cell carcinoma was the most commonly found histopathology. The majority of the subjects were diagnosed with stage IV cancer, and almost half of the subjects had nasopharyngeal cancer.

**Table 1:** Characteristic of the Study Participants (n = 85)

Variable	Result
**Age, years**	52.69 ± 12.52^*^
Age group < 60 years, n(%)	57 (67.1)
Age group ≥ 60 years, n(%)	28 (32.9)
**Gender, n(%)**	
Male	59 (69.4)
Female	26 (30.6)
**Body weight, kg**	54.35 ± 12.28^*^
**Body height, cm**	159.85 ± 8.50^*^
**BMI, kg/m** ^**2**^	21.16 ± 3.92^*^
**BMI classification, n(%)**	
Underweight	21 (24.7)
Normal	35 (41.2)
Overweight and obese	29 (34.1)
**Histopatology, n(%)**	
Squamous cell carcinoma	69 (81.2)
Adenocarcinoma	6 (7.1)
Mucoepidermoid	4 (4.7)
Others	6 (7)
**Cancer staging, n(%)**	
Stage I	1 (1.2)
Stage II	7 (8.2)
Stage III	30 (35.3)
Stage IV	47 (55.3)
**Cancer location, n(%)**	
Nasopharynx	41 (48.2)
Larynx	17 (20)
Mouth	10 (11.8)
Nose and paranasal sinuses	8 (9.4)
Salivary gland	5 (5.9)
Others	4 (4.7)
**Hemoglobin, g/dL**	12.07 ± 2.02^*^
**Thrombocyte, 10** ^**3**^ **/µL**	299 (37–617)^†^

BMI body mass index

^*^ mean ± SD

^†^ median (minimum-maximum)

### Total lymphocyte count

There were 70 (82.4%) subjects who had a normal TLC. Subjects with lower nutritional status had a lower level of TLC but showed no significant differences between other nutritional status groups. Higher TLC was found in male subjects and older adults. The TLC of the study participants is shown in Table 2.

**Table 2:** Total lymphocyte count of the study participants (n = 85)

Variable	Result	Mean difference (confidence interval)	p
**Total lymphocyte count, 10** ^**3**^ **/µL**	1.72 ± 0.74^*^		
Low TLC, n (%)	15 (17.6%)		
Normal TLC, n (%)	70 (82.4%)		
**Total lymphocyte count based on BMI, 10** ^**3**^ **/µL**			
Underweight (n = 21)	1.53 ± 0.61^*^		0.238^−^
Normal (n = 35)	1.68 ± 0.77^*^		
Overweight and obese (n = 29)	1.88 ± 0.78^*^		
**Total lymphocyte count based on gender, 10** ^**3**^ **/µL**			
Male (n = 59)	1.80 ± 0.76^*^	0.28 (0.06–0.62)	0.107
Female (n = 26)	1.52 ± 0.66^*^		
**Total lymphocyte count based on age classification, 10** ^**3**^ **/µL**			
Age group < 60 years (n = 57)	1.65 ± 0.76^*^	0.19 (0.17–0.53)	0.754
Age group ≥ 60 years (n = 28)	1.84 ± 0.70^*^		

BMI body mass index

TLC total lymphocyte count

^*^ mean ± SD

^s^ statistically significant (p < 0.05)

^−^ one-way ANOVA test, with LSD post hoc test: underweight vs. normal p = 0.452, underweight vs. overweight and obese p = 0.097, normal vs. overweight and obese p = 0.281

### Dietary intake

The median energy intake was 1505 (800–3040) kcal/day, while the protein intake of the participants was 73.96 ± 23.39 g/day. The BCAA intake was about 15% of the total protein intake. Data regarding the dietary intake of the subjects are shown in Table 3.

**Table 3:** Dietary Intake of the Study Participants (n = 85)

Variable	Result
**Energy intake, kcal/day**	1505 (800–3040)^†^
**Protein intake, g/dy**	73.96 ± 23.39^*^
**Non-protein calorie intake, kcal/day**	1297.36 ± 384.66^*^
**BCAA intake, g/day**	10.92 ± 4.39^*^
Leucine intake, g/day	4.72 ± 1.94^*^
Isoleucine intake. g/day	2.95 ± 1.23^*^
Valine intake. g/day	3.25 ± 1.27^*^

^*^ mean ± SD

^†^ median (minimum-maximum)

The comparison was made between the groups presented and are shown in Table 4. Subjects in the underweight groups showed the lowest BCAA intake among other groups. There was a significant comparison between male and female groups, in which males consumed more BCAA than females. Lower BCAA intake was found in participants aged less than 60 years old.

**Table 4:** Comparison of BCAA Intake of the Study Participants (n = 85)

Variable	Result	Mean difference (confidence interval)	p
BCAA intake based on BMI, g/day			
Underweight (n = 21)	9.53 ± 3.82^*^		0.197^−^
Normal (n = 35)	11.72 ± 4.19^*^		
Overweight and obese (n = 29)	10.95 ± 4.87^*^		
**BCAA intake based on gender, g/day**			
Male (n = 59)	11.61 ± 4.65^*^		0.040^s^
Female (n = 26)	8.35 (3.40–15.30)^†^		
**BCAA intake based on age classification, g/day**			
Age group < 60 years (n = 57)	10.58 ± 4.47^*^	1.02 (0.99–3.04)	0.315
Age group ≥ 60 years (n = 28)	11.60 ± 4.21^*^		

BCAA branched-chain amino acids

BMI body mass index

^*^ mean ± SD

^s^ statistically significant (p < 0.05)

^†^ median (minimum-maximum)

^−^ one-way ANOVA test, with LSD post hoc test: underweight vs. normal p = 0.072, underweight vs. overweight and obese p = 0.260, normal vs. overweight and obese p = 0.481

### Correlation between BCAA intake and TLC

Table 5 shows the correlation between BCAA intake and TLC, and was categorized into different categories. There was a statistically significant weak correlation between BCAA intake and TLC in the subjects. A medium correlation was found in the underweight, normal body mass index groups, and the older adults group. The BCAA intake did not show a significant correlation based on the gender classification groups.

**Table 5:** Correlation between BCAA Intake and TLC of the Study Participants (n = 85)

Variable	Total Lymphocyte Count	
	Correlation Coefficient (r)	P value
**BCAA intake (n = 85)**	+ 0.237	0.029^*^
Leucine	+ 0.236	0.029^*^
Isoleucine	+ 0.230	0.034^*^
Valine	+ 0.239	0.027^*^
**BCAA intake based on BMI**		
Underweight (n = 21)	+ 0.443	0.044^*^
Normal (n = 35)	+ 0.461	0.005^*^
Overweight and obese (n = 29)	-0.102	0.598
**BCAA intake based on gender**		
Male (n = 59)	+ 0.217	0.098
Female (n = 26)	+ 0.112	0.587
**BCAA intake based on age classification**		
Age group < 60 years (n = 57)	+ 0.150	0.264
Age group ≥ 60 years (n = 28)	+ 0.409	0.031^*^

BCAA branched-chain amino acids

BMI body mass index

^*^ statistically significant (p < 0.05)

Potential confounding variables related to the patients were identified, which were BMI, age and non-protein calorie. The only confounding factor that had no significant correlation with TLC was age; hence BMI and non-protein calorie intake were the two confounders included in the linear regression analysis. The analysis of potential confounders is shown in Table 6.

**Table 6:** Potential Confounding Variables (n = 85)

Variable	Total Lymphocyte Count	
	Correlation Coefficient (r)	P value
**BMI**	+ 0.183	0.093*
**Age**	+ 0.016	0.887
**Non-protein calorie intake**	+ 0.127	0.247*
**Hemoglobin**	+ 0.144	0.188*

BMI body mass index

^*^ statistically significant (p < 0.25)

The potential confounders and BCAA intake underwent further analysis using linear regression analysis. It is shown that there was no significant correlation between BCAA intake and total lymphocyte count after the adjustment. Table 7 shows the result of the analysis.

**Table 7:** Linear Regression Analysis of BCAA Intake, BMI, Gender, and TLC (n = 85)

Variable	Total Lymphocyte Count		
	Adjusted β	95% CI	p
**BCAA intake**	0.235	-0.001–0.080	0.056
**Non-protein calorie**	-0.047	-0.001-0.000	0.714
**BMI**	0.159	-0.012–0.072	0.162
**Hemoglobin**	0.108	-0.039-0.118	0.320

BCAA branched-chain amino acids

BMI body mass index

## Discussion

In this study, the correlation between BCAA and TLC was found to be insignificant after the adjustment for BMI, non-protein calorie intake, and hemoglobin. However, this study investigated the potential correlation between the BCAA intake and TLC in head and neck cancer patients who were underweight and had a normal nutritional status. This result is possible due to the relatively less BCAA intake in the underweight groups. Furthermore, the potential correlation was also found in older adults’ group. This result is also possible as older adults consume less BCAA than the other groups. Furthermore, this is a relative result that may made a bias indicating that the correlation between BCAA intake and TLC seems somewhat stronger than in the other groups.

The mean of BCAA intake of the subjects was 10.92 ± 4.39 g, with each type of amino acids intake were leucine intake of 4.72 ± 1.94 g, isoleucine intake of 2.95 ± 1.23 g, and valine intake of 3.25 ± 1.27 g. Among other BCAAs, leucine is a widely studied amino acid associated with lymphocyte activation [39]. Lower intake of BCAA was found mostly in participants less than 60 years old. Meanwhile, the lowest BCAA intake was found in the underweight groups among the other groups. Moreover, our analysis showed that male participants consumed significantly more BCAA than female participants. Based on national survey by BPS-Statistics Indonesia, a body of national survey, the protein intake of Indonesian people was 61.98 g [40], and was adequate as recommended by national adequacy standard of protein intake [41]. The subjects in this study have a higher protein intake with mean of 73.96 ± 23.39 g.

Our results from this study did not find a strong correlation between BCAA level and total lymphocyte counts in general. There are several things to be considered related to the different outcomes between this study and previous studies. Previous in vitro study have demonstrated that BCAA could improve the proliferation of lymphocytes, hence, has a strong correlation [42]. Several studies using cell culture observed that BCAA are a crucial component to the process of protein synthesis, deoxyribonucleic acid (DNA) and ribonucleic acid (RNA) replication in lymphocytes [43, 44]. However, study by Esposito et al. also reported that increasing BCAA concentrations extremely to 300% and 1000% above normal level did not give any effects on human T lymphocytes proliferation [23]. Although sounds really promising, most studies conducted so far were done only in animal models, limited evidences are available in human clinical trials. For instance, Polizzi et al. reported that BCAA, particularly leucine, may affect the mTOR signalling pathway in animal models. This is the pathway that is responsible for protein translation, proliferation, and cell growth in immune cells [15]. Polizzi et al. also reported their findings that T cells failed to proliferate normally when the mTOR pathway is defected [15, 45]. Chuang et al. was one of the first researchers to demonstrate the correlation between BCAA and lymphocyte count. Chuang et al. concluded from their study that lymphocyte proliferation is modulated by 15 amino acids in a culture medium, which is in ideal condition for optimal growth [42]. Another in vitro study in rats conducted by Tsukishiro et al. also confirmed the similar results. Tsukishiro et al. reported that BCAA administration has a stimulatory effect to lymphocyte proliferation in rats. Furthermore, these rats which were given BCAA have better outcomes in terms of immunity against liver cancer [46]. Similar findings were reported by Baakhtari et al. through their studies which indicated that BCAA supplementation has a positive impact on counteracting the immunosuppression mechanism in young racing horses [47]. Meanwhile, our study is a clinical trial in human that may not have the ideal condition for lymphocyte proliferation. The other reason for these differences is the amino acids other than BCAA that may have an effect on lymphocyte proliferation. From Chuang et al. study, it is reported that there are other essential amino acids that have correlation to lymphocyte proliferation [42]. An important mechanism suggested is that BCAA have a vital function in the process of protein synthesis through serving as a nitrogen donor for the synthesis process of glutamine. Glutamine is highly needed by the immune cells for proliferation and expression of surface markers [48].

On the other hand, when compared to previous clinical trial, particularly study by Bassit et al., this study also demonstrated that BCAA supplementation induced lymphocyte proliferation. However, this study was conducted in healthy individuals which still have a normal functioning immune system. In this study, there are changes in plasma glutamine level which is related to the decrease of lymphocyte proliferation after exercise. Nevertheless, Bassit et al. also found that in participants before doing exercise, BCAA supplementation does not improve lymphocyte proliferation [49]. Meanwhile, the clinical evidence between the correlation of BCAA and TLC in cancer patients is still rare. Most studies in cancer patients only measure the relation of BCAA levels to the risk of adverse outcomes [50]. Therefore, it can be concluded that there are several unexplainable correlations between the effect of the cancer itself to the metabolism of BCAA or the effect of the cancer to the lymphocyte proliferation. Moreover, further studies should also consider the effect of other amino acids which is not included as BCAA to the proliferation of lymphocytes.

This study has several strengths. Firstly, to our knowledge, this is one of the first clinical study assessing the correlation between BCAA intake and lymphocytes proliferation in cancer patients. Previous studies mostly done in healthy individuals which might have different metabolism process and immune system with cancer patients. Moreover, the results from this study are measured according to standardized and reliable methods systematically. However, this study still have several limitations. Firstly, this study could not consider the effect of other amino acids that is included in the protein intake of the participants. Further studies should be done with larger sample size and clearer protocol to be able to eliminate the probable effects of those amino acids which are not included as BCAA.

## Conclusion

This study has not demonstrated a significant correlation between BCAA intake from food sources and total lymphocyte counts. However, a strong correlation between BCAA intake and TLC has been found in underweight and older adults’ group. Branched-chain amino acids intake should still be considered for nutritional management in head and neck cancer patients due to its function in protein synthesis. Further studies should consider the role of non-BCAA amino acids in food source to the proliferation of lymphocytes. Moreover, further studies should be able to demonstrate the direct correlation of BCAA from blood serum to TLC in cancer patients.

## Data Availability

The datasets used and/or analysed during the current study are available from the corresponding author on reasonable request.

## List of abbreviations

AJCC: American Joint Committee on Cancer
BCAA: branched-chain amino acid
BMI: body mass index
DNA: deoxyribonucleic acid
FMUI: Faculty of Medicine, Universitas Indonesiaa
mTOR: mammalian target of the rapamycin
PNI: prognostic nutritional index
RNA: ribonucleic acid
SQ-FFQ: semi-quantitative food frequency questionnaire
TLC: total lymphocyte count
USDA: United States Department of Agriculture
